# Special Issue “*Bacillus subtilis* as a Model Organism to Study Basic Cell Processes”

**DOI:** 10.3390/microorganisms9122459

**Published:** 2021-11-28

**Authors:** Imrich Barák

**Affiliations:** Department of Microbial Genetics, Institute of Molecular Biology, Slovak Academy of Sciences, Dúbravská Cesta 21, 845 51 Bratislava, Slovakia; imrich.barak@savba.sk

*Bacillus subtilis* has served as a model microorganism for many decades. There are a few important reasons why this Gram-positive bacterium became a model organism to study basic cell processes. Firstly, *B. subtilis* has proven highly amenable to genetic manipulation and has become widely adopted as a model organism for laboratory studies. Secondly, it is considered as the Gram-positive equivalent of *Escherichia coli*, an extensively studied Gram-negative bacterium, and both microorganisms have served as examples for bacterial cell division studies in the last decades. Thirdly, unlike *E. coli*, it can form endospores and the sporulation represents the simplest cellular differentiation process. In addition, this bacterium became an exceptional specimen to study motility, chromosome segregation, competence, host system for bacteriophages, transcription regulation, biofilm formation, and *B. subtilis* is also widely used to produce various enzymes, such as proteases, amylase, etc.

The objective of this Special Issue on “*Bacillus subtilis* as a Model Organism to Study Basic Cell Processes” was to provide a platform to researchers for sharing their new studies on advances in basic cell processes studies in the model organism *B. subtilis*. The Special Issue includes papers from several leading scientists in the field studying sporulation, biofilm formation, bacteriophages, and transcription in this bacterium and its close relatives. The present Special Issue comprises nine research articles and two reviews.

Two articles were focused on studies of transcription and gene expression regulation in *B. subtilis*. Transcription of specific genes and production of physiologically relevant proteins is crucial for the adaptation of bacteria to changing environmental conditions. Sudzinová et al. [1] determined the role of DNA topology on transcription from rRNA promoters. Interestingly, they showed that the more negative DNA supercoiling in the exponential growth phase increases transcription from rRNA promoters. On the other hand, DNA relaxation in the stationary phase contributes to a decrease in their activity. This study points out the importance of DNA topology for the expression of rRNA and this is directly linked to nutrient availability. Vohradsky et al. [2] used an in silico approach with literature-mined data, gene expression modelling, and promoter sequence analysis to identify the regulon of *B. subtilis* sigma factor σ^B^ as a subunit of RNA polymerase. They concentrated on σ^B^-dependent genes expressed specifically during germination and the outgrowth of spores. They determined the binding motif of a subset of σ^B^ regulated genes during these parts of *B. subtilis* life cycle. Importantly, they also experimentally verified sixteen selected SigB dependant promoters.

Ermi et al. [3] studied the influence of Non-B DNA structures on stationary-phase mutagenesis in *B. subtilis*. They concentrated on G4 DNA and hairpin-forming motifs and they showed that these non-B DNA-forming structures promote genetic instability and may have spatial and temporal mutagenic effects. Faßhauer et al. [4] analyzed the role of two *B. subtilis* cold shock proteins, CspB and CspD proteins. These proteins belong to the most abundant proteins in the cell and thus it suggesting their possible crucial function. Deletion of either of the genes has no clear effect on phenotype. However, the simultaneous loss of both proteins results in severe growth effects and the appearance of suppressor mutations. Interestingly, the global RNA profile of the double mutant suggests that these proteins are important for transcription elongation and termination.

The next three articles are focused on different aspects of biofilm formation. *B. subtilis* and related bacteria belong to bacteria that can form a surface-associated multicellular assemblage, so-called biofilms. Dergham et al. [5] concentrated on the multi-culturing comparison between macrocolony, swarming, pellicle, and submerged biofilm of undomesticated *B. subtilis* NDmed strain. By using different 15 mutant strains they identified genes important for all biofilm phenotypes. Špacapan et al. [6] studied the role of quorum sensing protein ComX on biofilm formation. Their results indicate that ComX can mediate early commitment to sporulation and slow down biofilm formation. This quorum-sensing system can be important for modulating the coexistence of multiple biological states, biofilm formation, and sporulation. Stoll et al. [7] studied the surfactin production of two different *Bacillus velezensis* strains and analyzed its impacts on biofilm formation and stable colonization on different plant surfaces which finally enables its activity as an elicitor of induced systemic resistance.

*B. subtilis* has been especially well known for many decades of studies on sporulation, an important mechanism by which bacteria can survive harsh environmental conditions, and it also represents the simplest cell differentiation process. The spore resistance arises from several protective layers that surround the spore core. In addition to the cortex, a peptidoglycan layer, the coat is the main defense system against the challenges of the environment. More than 80 coat proteins are localized into four distinct morphological layers. Krajčíková et al. [8] investigated the interactions between SpoVM and SpoIVA and the proteins essential for cortex synthesis. They found and characterized their protein partners localized in the basement spore coat layer. Their results are linking the processes of cortex and coat formation. Tu et al. [9] analyzed the wet heat resistance of spores of *Bacillus subtilis* A163 in comparison with laboratory *B. subtilis*, PY79 strain. They showed extremely high resistance to wet heat compared to spores of laboratory strain. They determined the proteome of vegetative and sporulating cells of both strains A163 and PY79 and the results revealed proteomic differences of the two strains what should help to explain the high heat resistance of *B. subtilis* A163 spores.

This Special Issue also includes two review articles. Rismondo and Schulz [10] provided an overview of the alternative roles of ABC transporters affecting antibiotic resistance, cell wall biosynthesis, cell division, and sporulation in *B. subtilis* and *Listeria monocytogenes*. The authors focused on several ABC transporters, which are not only required to inactivate or export drugs but are essential for drug sensing, and on ABC transporters, which affect cell wall biosynthesis and remodeling. The second review by Łubkowska et al. [11] provides a reference compilation of 56 bacteriophages, which infect the species of thermophilic ‘*Bacillus* group’ bacteria. This review of bacteriophages of thermophiles is an important overview not only of their previously described characteristics but also their roles in many biogeochemical, ecological processes, and biotechnology applications, including emerging bionanotechnology.
